# Co-Microencapsulation of Sacha Inchi (*Plukenetia huayllabambana*) Oil with Natural Antioxidants Extracts

**DOI:** 10.3390/foods12112126

**Published:** 2023-05-25

**Authors:** Nancy Chasquibol, Billy Francisco Gonzales, Rafael Alarcón, Axel Sotelo, Gabriela Gallardo, Belén García, María del Carmen Pérez-Camino

**Affiliations:** 1 Grupo de Investigación en Alimentos Funcionales, Carrera de Ingeniería Industrial, Instituto de Investigación Científica, Universidad de Lima, Av. Javier Prado Este 4600, Fundo Monterrico Chico, Surco, Lima 15023, Peru; bgonzale@ulima.edu.pe (B.F.G.); ralarcor@ulima.edu.pe (R.A.); alex_94sc@hotmail.com (A.S.); 2 Instituto Nacional de Tecnología Agropecuaria (INTA), Gabriel de Aristizabal, B1686 William C. Morris, Buenos Aires C1033AAE, Argentina; gallardo.gabrielal@inta.gob.ar; 3 Instituto de la Grasa-Consejo Superior de Investigaciones Científicas, Campus Universidad Pablo de Olavide Ed. 46, Crtra. Sevilla-Utrera km 1, 41013 Sevilla, Spain; belgarpez@ipb.csic.es (B.G.);

**Keywords:** antioxidant, co-microencapsulation, skin, sacha inchi oil, camu camu, Andean potato, elderberry

## Abstract

Sacha inchi (*Plukenetia huayllabambana*) oil was co-microencapsulated with natural antioxidant extracts (NAE), such as camu-camu (*Myrciaria dubia* (HBK) Mc Vaugh) fruit, Añil variety Andean potato (*Solanum tuberosum* andigenum, and elderberry fruit (*Sambucus peruviana*). Gum Arabic and the ternary combination of gum Arabic (GA) + maltodextrin (MD) + whey protein isolate (WPI) at different formulations were used as coating materials for the encapsulation process using spray-drying. The moisture content, particle size distribution and morphology, total phenolic content, antioxidant activity, fatty acid and sterol composition, oxidative stability, and shelf-life were evaluated. Co-microcapsules of sacha inchi (*P. huayllabambana*) oil with camu camu skin extract (CCSE) at 200 ppm encapsulated with GA + MD + WPI had the highest total polyphenol content (4239.80 µg GAE/g powder), antioxidant activity (12,454.00 µg trolox/g powder), omega-3 content (56.03%), β-sitosterol (62.5%), greater oxidative stability (Oxidation Onset temperature of 189 °C), higher shelf-life (3116 h), and smaller particle sizes (6.42 μm). This research enhances the knowledge to obtain microcapsules containing sacha inchi (*P. huayllabambana*) oil with natural antioxidant extracts that could be used for the development of functional foods. Further research is needed to study the potential interactions and their influence between the bioactive components of the microcapsules and the challenges that may occur during scale-up to industrial production.

## 1. Introduction

Vegetable oil is an important source of polyunsaturated fatty acids (PUFAs) that are indispensable components of the human diet for being related to Non-Transmissible Chronic Diseases [1,2,3]. The variety of sacha inchi seeds, *Plukenetia huayllabambana* and *Plukenetia volubilis* (more commonly commercialized), produced a particularly high and appreciated content in PUFAs [4]. The essential fatty linolenic acid (omega-3) is the principal fatty acid in these two sacha inchi oil varieties, with percentages of 48% (*P. volubilis*) and 51% (*P. huayllabambana*). Alpha-linoleic and oleic acids are the second most important essential fatty acids of sacha inchi oil [5]. This exceptional essential fatty composition makes these oil oxidizable [6,7,8]. These characteristics could contribute to developing a rancid smell with displeasing taste and flavors [9]. On the other hand, there are relevant complications in food developing when oils are incorporated in distinct formulations because of their lower capability of being mixed in water; for that reason processing food powders containing edible oils that have a stable shelf-life during storage is a fast-growing area in the food industry [10].

Microencapsulation by spray-drying is one of the most practical and widespread ways to protect sacha inchi oil against oxidation. This process confers advantages in handling, release, and palatability of the microencapsulated product [11]. Microencapsulation improved solubility and the applicability of the oil in food matrices [12]. Chasquibol et al. [6] studied the glyceride and unsaponifiable compounds of microcapsules of sacha inchi (*P. huayllabambana* and *P. volubilis*) oils. Sacha inchi oil (*P. volubilis*) with combinations of skimmed milk powder, gum Arabic, and grape juice as coating materials were studied [13]. The use of Hi-cap^®^ and maltodextrin as encapsulating agents in microcapsules of this oil was also reported by Sanchez-Reinoso and Gutiérrez [8]. Pastuña-Pullutasig et al. [14] reported greater stability in the oxidation of *P. volubilis* oil microencapsulated with mixtures of gum Arabic and maltodextrin. Alarcón et al. [15] evaluated the stability and shelf life of *P. volubilis* and *P. huayllabambana* oil microencapsulated with Hi-Cap, gum Arabic, mixtures of gum Arabic (GA), maltodextrin (MD), and whey protein isolate (WPI). Landoni et al. [8] concluded that the different coating materials selected showed protection against oxidation in both species of sacha inchi oil microencapsulated. There are several studies where sacha inchi oil was microencapsulated; however, the oxidative stability and shelf-life could improve through the co-microencapsulation technique.

Co-encapsulation is an emerging field in the development of functional ingredients, whose approach is to have bioactive compounds, such as antioxidants, vitamins, oils, and others, in microcapsules to achieve synergistic effects between functional components [10,16]. Regarding co-encapsulated vegetable oils, less research has been reported than on fish oil. Sharif et al. [17] reported an increase in the oxidative stability of flaxseed oil microcapsules when co-microencapsulated with eugenol and β-carotenes. It also reported an efficient co-encapsulation of bioactive compounds, such as tocopherol (liposoluble) and naringenin (hydrosoluble), with WPI [18].

The aim of this research was to compare the physicochemical characterization, oxidation stability and shelf-life of the natural antioxidant extracts of camu camu skin (*Myrciaria dubia* (HBK) Mc Vaugh), elderberry fruit (*Sambucus peruviana*), and Andean potato skin (*Solanum tuberosum* andigenum Var. Añil) co-microencapsulated with sacha inchi (*Plukenetia huayllabambana*) oil (SIPHO) against synthetic antioxidant BHT at 200 ppm.

## 2. Materials and Methods

### 2.1. Raw Material

*P. huayllabambana* seeds were obtained in Rodriguez de Mendoza, region of Amazonas, Peru. Cold-pressed sacha inchi (*P. huayllabambana*) oil (SIPHO) was obtained in the Laboratorio de Alimentos Funcionales of the Universidad de Lima, Peru, and maintained at 4 °C until use. Potato variety Añil (*Solanum tuberosum* andigenum), Camu camu (*Myrciaria dubia* (HBK) Mc Vaugh), and elderberry (*Sambucus peruviana*) fruit were collected in the Peruvian regions of Huancavelica, Loreto, and Huánuco, respectively. Camu camu fruits, potato variety Añil, and elderberry fruit were grown in the districts of Saquena-Requena, Paucará-Acobamba, and Huánuco, respectively. The fruits and potatoes were washed, and their skins were freeze-dried (Alpha 1-2 LDplus, Martin Christ, Osterode am Harz, Germany) at 900 mmHg, −45 °C for 24 h. The lyophilized samples were stored in aluminized bags at freezing temperature.

### 2.2. Dry Skin Antioxidant Extracts

Freeze-dried samples of camu camu fruit, dried Añil potato skin, and elderberry fruit were pulverized in a processor (Grindomix GM200, Retsch, Haan, Germany) at a speed of 500 rpm for 5 min. Then, a weight of 2.50 g of ground sample was placed in a 600 mL beaker for every extract (lined with aluminum foil), and 200 mL of 50% (*v*/*v*) ethanol solution was added at pH 2 with 1.5 M HCl (Merck). It was left to stand for 12 h at refrigeration temperatures (Freezer Horizontal H420, Electrolux, Curitiba, Brazil), then it was removed until it reached room temperature (25 °C). The antioxidant compounds were extracted using sonifier equipment (Sonics Vibra Cell, Ningbo Kesheng Ultrasonic Equipment Co. Ltd., Ningbo, China) by the direct method at 100 W and 37 °C with constant agitation (VELP Scientifica, Usmate Velate, Italy) for 10 min; the process was repeated until obtaining a colorless sample. It was vacuum filtered (Trivac D16B, Leybold, Köln, Germany) with No. 2 filter paper and vacuum concentrated (R 100, BUCHI Labortechnic AG, Flawil, Switzerland) at a water bath temperature of 30 °C, speed 80 rpm, and a pump of 70 mbar pressure to eliminate the solvent. A concentrated extract was obtained and stored in an amber bottle at a temperature of −18 °C. Then, every extract was freeze-dried (Alpha 1-2 LDplus, Martin Christ, Osterode am Harz, Germany) at 900 mmHg, −45 °C for 24 h to obtain NAE: camu camu skin extract (CCSE), Añil variety potato skin extract (AVPSE), and elderberry fruit extract (EFE) powders. The NAE were kept in aluminized bags at −18 °C.

#### Total Phenolic Content (TPC) of Antioxidant Extracts

The TPC was performed by Folin–Ciocalteau assay [19,20] and Chasquibol et al. [21] with some modifications using a 1205 Vis Spectrophotometer (UNICO, Dayton, NJ, USA). All analyses were presented as mean values and performed in triplicate.

### 2.3. Co-Microencapsulation of SIPHO with NAE

Table 1 shows fourteen samples of formulations prepared with SIPHO and camu camu skin extract (CCSE), Añil variety potato skin extract (AVPSE), elderberry fruit extract (EFE) with gum Arabic (GA), maltodextrin (MD), whey protein isolate (WPI), and synthetic antioxidant BHT (200 ppm). The emulsions were prepared according to Landoni et al. [8] with some modifications. GA and the ternary mixture (GA:MD:WPI/3:13:3) were dispersed with distilled water under constant stirring overnight; then, the NAE (100 and 200 ppm) was incorporated regarding the oil content according to every formulation. The total solid concentration was adjusted at 30%. SIPHO was added at a concentration of 33.33% (1 g of oil/3 g of encapsulating agent) regarding total solids. A homogenizer Silverson (L5M-A, Silverson, Chesham, Buckinghamshire, England) was used to obtain homogenous emulsions at 9000 rpm for 10 min using a cooled bathwater to prevent emulsion temperature higher than 25 °C. The spray drying conditions were according to Chasquibol et al. [21] with some modifications using a spray-drier (Büchi B-290, Büchi Labortechnik AG, Flawil, Switzerland). The powders were stored in aluminized bags at −5 °C for later analysis.

#### 2.3.1. Moisture Determination

The moisture content was determined according to Alarcón et al. [22] with some modifications. The determinations were performed in triplicate.

#### 2.3.2. Particle Size Distribution and Morphology of Microcapsules

The particle size distribution and the microcapsule morphology were performed as Landoni et al. [8] reported with some modifications.

#### 2.3.3. Total Phenolic Content (TPC) and Surface Phenolic Content (SPC)

The TPC was determined by Folin–Ciocalteau assay [19,20] and Chasquibol et al. [21] with some modifications using a 1205 Vis Spectrophotometer (UNICO, Dayton, NJ, USA). Analyzes were performed in triplicate and presented as mean values.

The SPC was performed with 24 mg of microcapsules dissolved in 4.5 mL of CH_3_OH in a vortex for 1 min. The mixture was filtered with a Whatman filter paper N °2. The resulting solution was analyzed according to the previously described method. The efficiency of TPC microencapsulation (%) was calculated as follows (1):

(1) $$Polyphenol\;Encapsulation\;Efficiency\;(PEE)\;(\%)=\frac{\left(TPC-SPC\right)}{TPC}\times 100$$

#### 2.3.4. Determination of Antioxidant Activity on DPPH Radical

The antioxidant activity was determined by the DPPH method [23,24] and Chasquibol et al. [21] with some modifications using a 1205 Vis Spectrophotometer (UNICO, Dayton, NJ, USA). All assays were performed in triplicate. The percentage of inhibition (% I) of free radicals was determined as follows (2):

(2) $$(\%I)=\frac{\left(Abs517\;control)-(Abs517\;sample\right)}{Abs517\;control}\times 100$$

#### 2.3.5. Fatty Acid Composition

Fatty acids methyl esters (FAMEs) were made according to Chasquibol et al. [21] with some modifications through 7890B Agilent gas chromatograph (GC 7890B Agilent Technologies, Santa Clara, CA, USA) equipped with an SP2380 polar capillary column (poly (90% biscyanopropyl-10% cyanopropylphenyl) siloxane, 60 m × 0.25 mm i.d.; 0.20 µm film thickness), and a flame ionization detector. The injector and detector temperatures were adjusted at 225 and 250 °C, respectively. The flow rate of the carrier gas (hydrogen) was 1.0 mL/min. The oven temperature was fixed at 165 °C and increased to 230 °C at 3 °C/min, maintaining this temperature for 2 min. The injection volume was 1 µL in split mode, and the determination was conducted by duplicate for each sample.

#### 2.3.6. Sterol Composition

Starting from the oils extracted from the microcapsules obtained according to the previous section, the procedure described by García-González et al. [25] was followed, with a slight modification that consisted of avoiding passage through an amino column. A total of 50 mg was weighed, to the nearest mg, in a 30 mL capacity test tube with a screw cap. Prior to weighing the sample, 100 µL of an α-cholestanol solution was added to the tube as an internal standard for the quantitative determination of sterols. The solvent was removed to dry under a stream of nitrogen. Next, 5 mL of 3% sodium methylate was added, and the tube was kept closed in a bath at 80 °C for 30 min. After this time, 5 mL of a 4% sulfuric/methanol solution was added, and heating was continued for 30 min. Then, it was allowed to cool, and 2 mL of aqueous saline solution (saturated sodium chloride water) was added and extracted 3 times with 2 mL of hexane each time. The hexane extracts were received in a test tube and washed 2 times with 2 mL of water. After the washings, two spatulites of anhydrous sodium sulfate were added, and the clear solution was transferred to another tube, where it was brought to dryness under a stream of nitrogen. It was then derivatized by adding 300 µL of silanizing reagent (BSTFA-TMCS, 99:1) and injected into gas chromatography. The chromatographic conditions were the following: HP-5 column (30 m × 0.25 I.D. × 0.1 μm); oven temperature: isotherm at 265 °C; injector and detector: 300 °C and, 1 mL was injected in split mode. Split injection system.

#### 2.3.7. Oxidative Stability and Shelf-Life

The oxidative stability of microcapsules was evaluated according to Chasquibol et al. [21] with some modifications. The Oxidation Onset Temperature (OOT) was determined using the ASTM E2009-08 Method A through Differential Scanning Calorimetry (Mettler 20, Mettler Toledo, Columbus, OH, USA), and the test was carried out in duplicate. The Rancimat test was performed according to Velasco et al. [26] in an 892 Professional Rancimat^©^ (Metrohm, Herisau, Switzerland). The shelf life at 25 °C was calculated according to Chasquibol et al. [21].

### 2.4. Statistical Analysis

Results were presented as mean ± standard deviation. Assays were carried out in duplicate or triplicate. Analysis of variance (ANOVA) and Tukey’s test were performed at a 95% of significance level with Minitab 18.0 (Minitab^®^ statistical software, State College, PA, USA).

## 3. Results

### 3.1. Total Polyphenolic Content of Dried Skins

The TPC of camu camu skin was higher (124. 37 ± 0.18 mg GAE/g powder) compared to those reported by Montero Fernandez et al. (12.41 mg GAE/g) [27] and Conceição et al. (33.4 mg/g) [28], but slightly close to Neves et al. (148.54 mg GAE/g) [29], which analyzed the TPC value after eighty-eight days of the onset of anthesis of the inflorescences in the field. It is worth noting that camu camu samples were collected from a specific town called Bagazán in Loreto, Peru. No studies have yet been found on camu camu from the town of Bagazán. The TPC of potato variety Añil skin was higher (73,838.80 ± 4453. 92 µg GAE/g powder) than reported by Bellumori et al. (14.53 mg of phenolic acids/g) [30], performed by HPLC–PDA–MS, while the TPC of elderberry fruit was higher (134,173.10 ± 5050.40 µg GAE/g powder) than the TPC reported by Chirinos et al. (33.2 mg GAE/g DW) [31]. Due to the variations in cultivation conditions, extraction parameters and analytical methods could have proffered to the diversity of polyphenolic amounts. For instance, the major polyphenols for camu camu skin are common derivatives of the ellagic acid [32]; for potato variety Añil skin are habitual 5-caffeoyquinic acid, 4-caffeoyquinic acid, caffeic acid, and cinnamic acid [30], and for elderberry fruit are chlorogenic acid, epicatechin, catechin, p-coumaric acid, and caffeic acid [33].

### 3.2. Moisture Determination and Particle Size Distribution

According to Table 2, the moisture content ranged between 2.59 ± 0.73% to 7.79 ± 0.04%; these were similar to the typical values found in spray-dried powders. It was observed that microcapsules with antioxidant extracts at 200 ppm showed lower moisture content than microcapsules at 100 ppm, which was higher than what was described by Souza et al. [34] and Comunian et al. [35]. Souza et al. [34] reported that WPI could increase the moisture content in microcapsules because this component makes it difficult to remove water from the particles during drying.

Regarding particle size distribution (Table 2), the *D* [4, 3] of microcapsules was between 4.17 to 20.89 μm for formulations with GA, being the highest value for microcapsules with CCSE (200 ppm), whereas the formulations with the ternary mixture (GA + MD + WPI), the *D* [4, 3] was between 6.42 to 12.41 μm, being the highest value for microcapsules with BHT (200 ppm). A monodisperse distribution was shown in all formulations. In this research, the incorporation of antioxidants extracts could increase the *D* [4, 3] value; all microcapsules with antioxidant extracts described higher values than those reported (SIPHO + GA, and SIPHO + GA + MD + WPI) by Landoni et al. [16]. Vishnu et al. [36] found a particle size of 2.3 μm for sardine oil with vanillic acid as an antioxidant agent. Binsi et al. [37] reported particle sizes around 115 nm with salvia polyphenol extract in sardine oil microcapsules.

### 3.3. Morphology Analysis of Microcapsules

**Figure 1:**
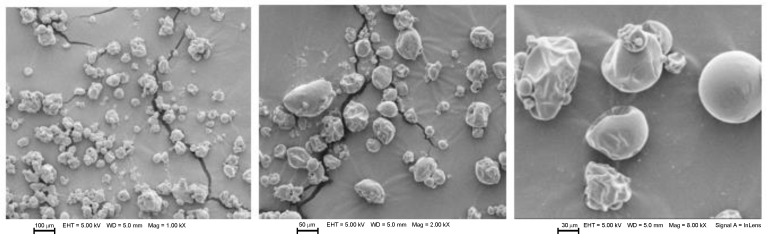
SEM micrographs from microencapsulated *P. huayllabambana* oil (SIPHO) + GA + MD + WPI + BHT (200 ppm). EHT: Electron high tension. WD: Working distance. Mag: Magnification.

**Figure 2:**
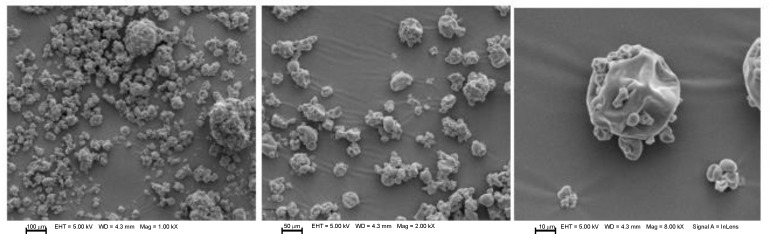
SEM micrographs from microencapsulated *P. huayllabambana* oil (SIPHO) + GA + MD + WPI + AVPSE (200 ppm). EHT: Electron high tension. WD: Working distance. Mag: Magnification.

**Figure 3:**
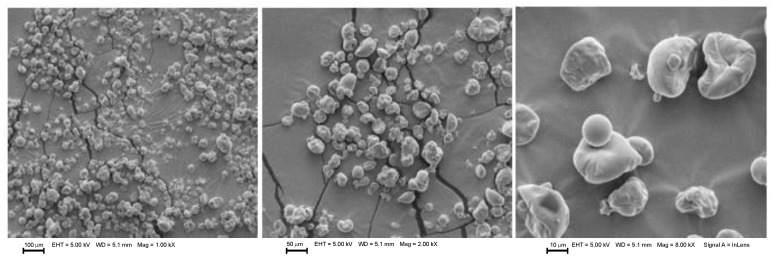
SEM micrographs from microencapsulated *P. huayllabambana* oil (SIPHO) + GA + MD + WPI + CCSE (200 ppm). EHT: Electron high tension. WD: Working distance. Mag: Magnification.

**Figure 4:**
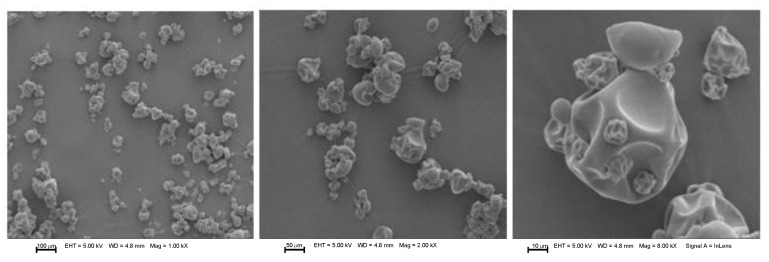
SEM micrographs from microencapsulated *P. huayllabambana* oil (SIPHO) + GA + MD + WPI + EFE (200 ppm). EHT: Electron high tension. WD: Working distance. Mag: Magnification.

The morphological assay performed by scanning electron microscopy (SEM) can be observed in Figure 1, Figure 2, Figure 3 and Figure 4 for microcapsules with GA + MD + WPI and NAE at 200 ppm. In general, the microcapsules present shapes from round to partially collapsed with cracks and low agglomeration. It can be distinguished that the BHT and microcapsules with camu camu extracts had rounded shapes, unlike the elderberry and Añil potato samples, where their microcapsules have a spherical shape with cracks or crater-like fissures, which could indicate that the samples with BHT and camu camu could result in higher oxidative stability. The morphological differences and collapsed structures of microcapsules were an expected behavior for samples produced by spray-drying.

Aghbashlo et al. [38] stated that microcapsules with a spherical shape and without fissures or cracks protect the encapsulated oil from oxidation by preventing it from migrating to the surface and preventing air from penetrating it. This is consistent with the rounded shape reported in the samples by Comunian et al. [35], Vishnu et al. [36], Yeşilsu, and Özyurt [39], who microencapsulated fish oil with rosemary, thymol, and laurel extracts and obtained rounded microcapsules without cracks or craters.

### 3.4. Total Phenolic Content (TPC) and Surface Phenolic Content (SPC) of Microcapsules

The TPC values are shown in Table 3. The microcapsules formulated with camu camu skin extract had the highest TPC and SPC value than other formulations. The polyphenolic encapsulation efficiency (PEE) ranged from 56.34 ± 6.83% for SIPHO + GA + BHT (200 ppm) to 95.64 ± 2.16% for SIPHO + GA + MD + WPI + AVPSE (200 ppm). The highest amount of the PEE and the lowest contents of the SPC could indicate that the phenolics compounds from the antioxidant extracts were efficiently encapsulated and incorporated into the core of the microcapsule. Therefore, the spray drying procedure was efficient in encapsulating polyphenol extract ingredients into the core. The results indicated that camu camu antioxidant extracts are better than BHT principally due to their higher composition of polyphenolic compounds such as ellagic acid, myricetin, and others [32]. In addition, microcapsules formulated with WPI were higher regarding TPC values. This could be explained due to proteins that can modify emulsions by changing their surface tension; thus, this strengthens the core and encapsulating agent interactions [40]. That is to say that protein-based coating formulations could efficiently maintain the polyphenolics compounds inside the core [41].

### 3.5. Antioxidant Activity of Microcapsules

As shown in Table 4, all the microcapsules with natural antioxidants extract showed higher antioxidant activity than the formulation coated with BHT in the maximum allowed concentration (200 ppm) according to current legislation. The highest values of antioxidant activity were for the microcapsules formulated with SIPHO + GA + MD + WPI + AVPSE (200 ppm) (16,430.20 ± 104.20 µg Trolox/g powder) and SIPHO + GA + MD + WPI + AVPSE (100 ppm) (13,590.50 ± 29.00 µg Trolox/g powder), followed by the SIPHO+ GA + CCSE (200 ppm) (12,716.00 ± 241.00 µg Trolox/g powder). The inhibition (%) are ranged from 51.28 ± 1.37 I% for SIPOH + GA + MD + WPI + BHT (200 ppm) to 99.66 ± 0.29 I% for SIPHO + GA + MD + WPI + ACPSE (200 ppm). For Bellumori et al. [30], the Andean Añil potato variety reported 1453.22 mg/100 g of phenolic acids, which could be the cause of the higher antioxidant activity in the microcapsules with Añil potato skin extracts. Moreover, the great antioxidant activity of co-microcapsules formulated with antioxidant extracts could be partly explained by the sterol compounds present in SIPHO; all microcapsules have been loaded with SIPHO. Cheng et al. [42] mentioned that sterol compounds are natural liposoluble compounds with antioxidant activity.

### 3.6. Fatty Acid Composition of Microcapsules

Table 5 shows the conservation of polyunsaturated ω-3 fatty acids as the values are close to the initial oil sample. It should be noted the absence of trans isomers in all the samples analyzed, except in the microcapsules containing camu camu at concentrations of 100 and 200 ppm. Since this fact was not repeated when this same oil was used, with the same encapsulating agents, but without the antioxidants, as shown by Chasquibol et al. [5], it could be explained that the formation of the trans isomer could have been due to the fact that the water elimination process has been carried out more slowly in this case, with which the oil has been exposed to the working temperature for a longer time.

### 3.7. Sterol Analysis of Microcapsules

The sterol compositions are presented in Table 6. The Cholesterol was present in the encapsulating agent containing WPI. Therefore, the WPI wall material should be dispensed in future food formulations since healthy food should not be prepared to contain cholesterol in the amounts quantified (~1000 mg/kg of oil) in this study. The average data obtained for these kinds of lipids of the microencapsulated agree with those corresponding to the starting oils, which would indicate that the sterols of the sacha inchi oils are fully available after the encapsulation process, as they are in oils. Regarding the special relationship between stigmasterol and campesterol observed in these oils (average of the evaluated oils: 5.96 *P. huayllabambana*), an average of the same order as that found in the respective oils is observed for the sterols from the microencapsulated with *P. huayllabambana* (5.13). Among the phytosterols in the microcapsules (from the oils), the majority was β-sitosterol (~60.6% in *P. huayllabambana* microcapsules).

### 3.8. Oxidation Stability and Shelf-Life of Microcapsules

The oxidation stability of microcapsules is reported in Table 7. All microcapsules with antioxidants extracts were better in comparison to microcapsules with BHT at 200 ppm. In addition, the oxidation stability of SIPHO microcapsules with natural antioxidants was greater than the SIPHO (169 °C) [8]. The OOT of the camu camu microcapsules reported the highest values (187 to 189 °C). This may be due to the great antioxidant capacity and polyphenols content that this Amazonian fruit has [27,43,44] better morphological and size distribution characteristics that have been detailed above. The promoting behavior of the oxidative stability in oil microcapsules can also be observed in what Yeşilsu and Özyurt [39] reported, which mentioned that extracts of rosemary at 1500 ppm are more stable than with BHT at 250 ppm. A similar protective or lipid oxidation reduction effect can be observed in fish oil microcapsules with the incorporation of oregano extracts [45].

**Figure 5:**
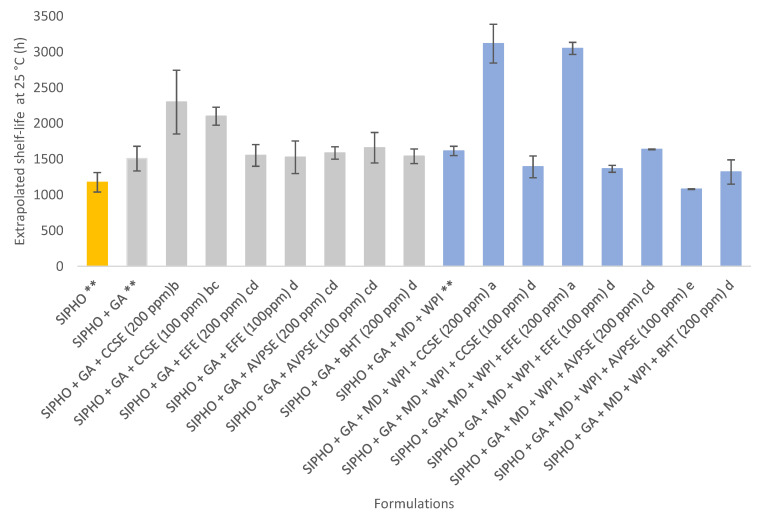
Shelf-life of SIPHO microencapsulated with NAE. Results are expressed as means ± SD (*n* = 3). ^a,b,c,d,e,^ values in the same column with different letters differ significantly when *p* < 0.05. ** Data available on [8].

The microcapsules with natural antioxidants of camu camu, elderberry, and Añil potato had higher shelf-life values (Figure 5). Regarding microcapsules with GA and GA + MD + WPI as encapsulating agents, there is no clear difference in shelf life between formulations with natural antioxidants and BHT, except for the SIPHO + GA + CCSE (200 ppm) (2298.00 ± 446.00 h), SIPHO + GA + MD + WPI + EFE (200 ppm) (3049.60 ± 84.50 h), and SIPHO + GA + MD + WPI + CCSE (200 ppm) (3116.00 ± 271.0 h). This could be due to the specific sterol composition of the SIPHO (Table 6) and the presence of the phenolic compounds with antioxidant activity (Table 4) of the camu camu skin and elderberry fruit. These results agreed with the Oxidation Onset temperature (OOT) data shown in Table 7, where the great oxidation stability of camu camu antioxidants was confirmed. In resume, all microcapsules with antioxidant extracts showed better shelf-life at 25 °C (h) than SIPHO (1176 ± 136.8 h) reported by Landoni et al. [8]. These results prove that the spray-drying procedure was suitable to effectively co-microencapsulate together both sacha inchi oil and natural antioxidant extracts.

## 4. Conclusions

The co-microencapsulation process of sacha inchi (*P. huayllabambana*) oil with natural antioxidants extracts showed better oxidative stability and shelf-life than microcapsules with BHT at 200 ppm. Formulations with NAE had great antioxidant activity (9366.10 ± 31.10–116,430.20 ± 104.20 µg trolox/g powder) due to their high sterols content, principally β-sitosterol (53.8 ± 1.0%–66.6 ± 1.0%) and total phenolic content (501.48 ± 13.96–4239.80 ± 26.40 µg GAE/g powder). All formulations showed a high percentage of omega-3 (55.83 ± 0.64–58.36 ± 0.85%). It can be highlighted that the oxidative stability (189 °C) and shelf-life (3116 h) of microcapsule of SIPHO with Camu camu skin extract at 200 ppm coated with GA + MD + WPI was superior to BHT at 200 ppm (178 °C, and 1320 h), so the camu camu skin extract could be used as a natural antioxidant. Therefore, the co-microencapsulation of sacha inchi (*P. huayllabambana*) oil with natural antioxidant extracts has better protection against oxidation compared with the synthetic antioxidant, BHT. Further research is needed to study the potential interactions and their influence between the bioactive components of the microencapsulation of sacha inchi (*P. huayllabambana*) oil with natural antioxidants extracts and their scale-up to industrial production.

## Figures and Tables

**Table 1:** Formulations of microencapsulated SIPHO with NAE.

Formulations	Microencapsulated SIPHO with NAE Formulations
1	SIPHO + GA + CCSE (200 ppm)
2	SIPHO + GA + CCSE (100 ppm)
3	SIPHO + GA + EFE (200 ppm)
4	SIPHO + GA + EFE (100 ppm)
5	SIPHO + GA+ AVPSE (200 ppm)
6	SIPHO + GA+ AVPSE (100 ppm)
7	SIPHO + GA +BHT (200 ppm)
8	SIPHO + GA + MD + WPI + CCSE (200 ppm)
9	SIPHO + GA + MD + WPI + CCSE (100 ppm)
10	SIPHO + GA +MD +WPI+ EFE (200 ppm)
11	SIPHO + GA +MD +WPI+ EFE (100 ppm)
12	SIPHO + GA + MD + WPI + AVPSE (200 ppm)
13	SIPHO + GA+ MD + WPI + AVPSE (100 ppm)
14	SIPHO + GA + MD + WPI + BHT (200 ppm)

SIPHO: Sacha inchi (*Plukenetia huayllabambana*) oil. CCSE: Camu camu skin extract. EFE: Elderberry fruit extract. AVPSE: Añil variety potato skin extract. BHT Butylated hydroxytoluene.

**Table 2:** Moisture content (%)and particle size (µm) of SIPHO microencapsulated with NAE.

Microencapsulated SIPHO with NAE Formulations	Moisture Content (%)	*D* [4, 3] µm	Span	Volume Distribution µm		
				*D* (*v*, 0.1)	*D* (*v*, 0.5)	*D* (*v*, 0.9)
SIPHO + GA **	5.87 ± 0.13	2.6	2.0	0.8	2.1	5.1
1	5.53 ± 0.04 ^d,e^	20.89	1.055	1.12	6.19	66.40
2	6.17 ± 0.04 ^c,d^	-	-	-	-	-
3	2.59 ± 0.73 ^h^	4.17	2.614	0.96	3.09	9.03
4	6.60 ± 0.13 ^c^	-	-	-	-	-
5	6.71 ± 0.01 ^b,c^	5.88	2.798	0.92	3.08	9.54
6	7.79 ± 0.04 ^a^	-	-	-	-	-
7	4.35 ± 0.21 ^f,g^	-	-	-	-	-
SIPHO + GA + MD + WPI **	3.58 ± 0.09	6.1	2.9	0.9	3.2	10.1
8	4.00 ± 0.14 ^g^	6.42	2.146	1.18	4.69	11.24
9	6.28 ± 0.13 ^c,d^	7.47	2.360	1.27	5.68	14.66
10	4.18 ± 0.11 ^f,g^	10.19	2.071	1.76	8.90	20.19
11	3.53 ± 0.04 ^g^	-	-	-	-	-
12	7.50 ± 0.03 ^a,b^	7.08	2.262	1.32	5.88	14.62
13	7.59 ± 0.12 ^a^	-	-	-	-	-
14	4.94 ± 0.05 ^e,f^	12.41	2.089	2.08	9.60	22.14

Results are expressed as means ± SD (*n* = 3). ^a,b,c,d,e,f,g,h^ values in the same column with different letters differ significantly when *p* < 0.05. *D* [4, 3] µm: volume-weighted mean size. *D* (*v*, 0.1): particle size for which 10% of the sample is less than that limit. *D* (*v*, 0.5): particle size for which 50% of the sample is less than that limit. *D* (*v*, 0.9): particle size for which 90% of the sample is less than that limit. Span: dispersion index. ** Data available on [8]. For formulations 1–14, review Table 1.

**Table 3:** TPC (µg GAE/g powder), SPC (µg GAE/g powder), PEE (%) of SIPHO microencapsulated with NAE.

Microencapsulated SIPHO with NAEFormulations	TPC (µg GAE/g Powder)	SPC (µg GAE/g Powder)	PEE (%)
1	3780.27 ± 14.38 ^b^	633.70 ± 18.60 ^c^	86.72 ± 0.60 ^b,c,d^
2	3245.00 ± 66.60 ^c^	775.20 ± 35.70 ^a^	79.69 ± 1.55 ^d,e^
3	757.70 ± 8.56 ^j^	107.50 ± 17.70 ^g^	92.81 ± 2.56 ^a,b^
4	589.70 ± 67.20 ^k^	114.80 ± 31.10 ^g^	85.61 ± 1.54 ^c,d,e^
5	976.72 ± 11.94 ^i^	196.46 ± 1.62 ^d^	88.97 ± 0.94 ^a,b,c^
6	522.27 ± 14.26 ^k^	79.48 ± 4.48 ^g^	61.93 ± 1.35 ^f^
7	501.48 ± 13.96 ^k^	213.90 ± 34.70 ^d^	56.34 ± 6.83 ^f^
8	4239.80 ± 26.40 ^a^	621.20 ± 22.00 ^c^	88.62 ± 0.64 ^a,b,c^
9	2823.80 ± 33.50 ^d^	710.10 ± 20.90 ^b^	79.06 ± 1.36 ^e^
10	2235.00 ± 35.90 ^f^	132.28 ± 8.91 ^e,f,g^	93.43 ± 0.29 ^a,b^
11	1970.00 ± 31.60 ^g^	182.23 ± 0.44 ^d,e,f^	93.49 ± 0.10 ^a,b^
12	1876.24 ± 14.17 ^g^	188.08 ± 7.95 ^d,e^	95.64 ± 2.16 ^a^
13	1478.60 ± 25.30 ^h^	225.95 ± 12.37 ^d^	82.77 ± 3.09 ^c,d,e^
14	2566.30 ± 99.50 ^e^	123.50 ± 15.67 ^f,g^	94.61 ± 0.42 ^a^

Results are expressed as means ± SD (*n* = 3). ^a,b,c,d,e,f,g,h,i,j,k^ values in the same column with different letters differ significantly when *p* < 0.05. For formulations 1–14, review Table 1.

**Table 4:** Antioxidant activity DPPH (µg trolox/g dm) and inhibition (%) of SIPHO microencapsulated with NAE.

Microencapsulated SIPHO with NAEFormulations	DPPH (µg trolox/g Powder)	Inhibition (%)
1	12,716.00 ± 241.00 ^c^	78.97 ± 0.67 ^b^
2	11,270.00 ± 260.00 ^d^	69.82 ± 0.55 ^i^
3	10,353.00 ± 182.00 ^e,f^	64.41 ± 0.23 ^f,g^
4	10,137.90 ± 45.80 ^f^	63.81 ± 0.31 ^g,h^
5	12,769.90 ± 129.60 ^c^	73.70 ± 0.77 ^d,e,f^
6	11,458.50 ± 1.00 ^d^	69.01 ± 0.77 ^g,h^
7	10,608.00 ± 204.00 ^e^	70.93 ± 1.28 ^h^
8	12,454.00 ± 183.00 ^c^	77.36 ± 0.38 ^c,d,e^
9	11,451.40 ± 77.40 ^d^	71.72 ± 0.13 ^b,c^
10	10,691.12 ± 111.00 ^e^	65.71 ± 0.26 ^b,c,d^
11	9366.10 ± 31.10 ^g^	58.40 ± 0.09 ^e,f^
12	16,430.20 ± 104.20 ^a^	99.66 ± 0.29 ^a^
13	13,590.50 ± 29.00 ^b^	73.70 ± 0.77 ^d,e,f^
14	7408.00 ± 235.00 ^h^	51.28 ± 1.37 ^j^

Results are expressed as means ± SD (*n* = 3). ^a,b,c,d,e,f,g,h,i,j^ values in the same column with different letters differ significantly when *p* < 0.05. For formulations 1–14, review Table 1.

**Table 5:** Composition in fatty acids (%) of SIPHO microencapsulated with NAE.

Microencapsulated SIPHO with NAEFormulations	C_16:0_	C_16:1_	C_17:0_	C_17:1_	C_18:0_	C_18:1_	C_18:2_	C_20:0_	C_18:3_	C_20:1_	*trans*
SIPHO	4.50 ± 0.24 ^bcd^	0.07 ± 0.01	0.06 ± 0.01	0.04 ± 0.01	1.75 ± 0.05 ^abcd^	7.95 ± 0.59 ^b^	26.10 ± 0.50 ^ab^	0.31 ± 0.15	58.17 ± 0.55	0.29 ± 0.15 ^ab^	nd
1	4.72 ± 0.28 ^abcd^	0.07 ± 0.01	0.06 ± 0.01	0.04 ± 0.01	1.82 ± 0.06 ^abcd^	8.77 ± 0.20 ^ab^	26.97 ± 0.88 ^ab^	0.29 ± 0.07	56.98 ± 0.57	0.19 ± 0.07 ^b^	0.07 ± 0.01
2	4.75 ± 0.14 ^abcd^	0.06 ± 0.11	0.06 ± 0.01	0.05 ± 0.01	1.85 ± 0.07 ^abcd^	8.79 ± 0.21 ^ab^	26.98 ± 0.81 ^ab^	0.29 ± 0.07	56.95 ± 0.85	0.13 ± 0.07 ^b^	0.07 ± 0.01
3	4.46 ± 0.27 ^bcd^	nd	0.05 ± 0.00	nd	1.67 ± 0.11 ^bcd^	8.54 ± 0.21 ^ab^	26.80 ± 0.72 ^ab^	0.26 ± 0.12	58.07 ± 0.82	0.16 ± 0.07 ^b^	nd
4	4.33 ± 0.14 ^cd^	0.04 ± 0.00	0.05 ± 0.00	nd	1.58 ± 0.07 ^d^	8.43 ± 0.28 ^ab^	26.45 ± 0.64 ^ab^	0.25 ± 0.11	58.36 ± 0.85	0.45 ± 0.06 ^a^	nd
5	4.46 ± 0.21 ^bcd^	0.06 ± 0.01	0.07 ± 0.01	0.04 ± 0.01	1.61 ± 0.10 ^cd^	8.13 ± 0.52 ^b^	28.72 ± 0.71 ^a^	0.26 ± 0.10	56.45 ± 0.78	0.18 ± 0.03 ^b^	nd
6	4.23 ± 0.10 ^d^	0.06 ± 0.01	0.06 ± 0.01	0.04 ± 0.01	1.63 ± 0.14 ^cd^	8.22 ± 0.68 ^b^	28.73 ± 0.85 ^a^	0.26 ± 0.06	56.57 ± 0.88	0.19 ± 0.04 ^b^	nd
7	4.50 ± 0.21 ^bcd^	0.04 ± 0.00	0.06 ± 0.00	nd	1.72 ± 0.10 ^abcd^	8.54 ± 0.37 ^ab^	26.67 ± 0.52 ^ab^	0.27 ± 0.10	57.82 ± 0.99	0.37 ± 0.08 ^ab^	nd
8	5.20 ± 0.24 ^ab^	0.09 ± 0.01	0.07 ± 0.01	0.04 ± 0.01	2.03 ± 0.08 ^ab^	9.25 ± 0.24 ^ab^	26.72 ± 0.71 ^ab^	0.29 ± 0.07	56.03 ± 0.66	0.16 ± 0.03 ^b^	0.07 ± 0.01
9	5.05 ± 0.17 ^abc^	0.08 ± 0.01	0.07 ± 0.01	0.04 ± 0.01	1.96 ± 0.08 ^abc^	9.00 ± 0.42 ^ab^	25.82 ± 0.57 ^b^	0.29 ± 0.11	56.41 ± 0.71	0.16 ± 0.03 ^b^	0.07 ± 0.01
10	5.40 ± 0.13 ^a^	0.04 ± 0.00	0.05 ± 0.00	0.04 ± 0.00	1.85 ± 0.10 ^abcd^	8.8 ± 0.23 ^ab^	27.00 ± 0.78 ^ab^	0.27 ± 0.11	57.10 ± 0.95	0.30 ± 0.08 ^ab^	nd
11	5.20 ± 0.16 ^ab^	0.06 ± 0.00	0.07 ± 0.00	nd	1.93 ± 0.08 ^abcd^	8.95 ± 0.29 ^ab^	26.36 ± 0.72 ^ab^	0.26 ± 0.10	56.81 ± 0.75	0.34 ± 0.03 ^ab^	nd
12	5.21 ± 0.21 ^ab^	0.09 ± 0.01	0.07 ± 0.01	0.04 ± 0.01	2.02 ± 0.07 ^ab^	9.14 ± 0.48 ^ab^	26.76 ± 0.64 ^ab^	0.29 ± 0.07	56.20 ± 0.85	0.16 ± 0.03 ^b^	nd
13	5.31 ± 0.18 ^a^	0.10 ± 0.01	0.07 ± 0.01	0.04 ± 0.01	2.05 ± 0.08 ^a^	9.43 ± 0.41 ^ab^	26.65 ± 0.78 ^ab^	0.29 ± 0.08	55.89 ± 0.86	0.14 ± 0.01 ^b^	nd
14	5.42 ± 0.20 ^a^	0.08 ± 0.01	0.07 ± 0.01	0.04 ± 0.01	1.97 ± 0.13 ^abc^	9.91 ± 0.66 ^a^	26.32 ± 0.71 ^ab^	0.18 ± 0.01	55.83 ± 0.64	0.17 ± 0.01 ^b^	nd

nd = not detected. ^a,b,c,d^ values in the same column with different letters differ significantly when *p* < 0.05. For formulations 1–14, review Table 1.

**Table 6:** Composition in sterols (mg/kg) of SIPHO microencapsulated with NAE.

Microencapsulated SIPHO with NAE Formulations	Total Plant Sterols (mg/kg)	β-Sitosterol (Phytosterol Majority) (%)	Stigmasterol/Campesterol Ratio	Cholesterol (mg/kg)
SIPHO	1856 ± 124 ^c^	56.7 ± 1.5 ^def^	5.96 ± 0.63 ^a^	nd
1	2411 ± 106 ^a^	60.1 ± 1.4 ^bcde^	4.62 ± 0.68 ^ab^	nd
2	2235 ± 113 ^abc^	59.8 ± 1.1 ^bcde^	5.57 ± 0.57 ^ab^	nd
3	1966 ± 115 ^bc^	61.8 ± 1.0 ^bc^	5.44 ± 0.59 ^ab^	nd
4	1987 ± 100 ^abc^	61.2 ± 1.1 ^bcde^	5.03 ± 0.58 ^ab^	nd
5	2187 ± 105 ^abc^	56.5 ± 1.0 ^ef^	5.76 ± 0.62 ^a^	nd
6	2173 ± 103 ^abc^	57.4 ± 1.4 ^cdef^	4.31 ± 0.55 ^ab^	nd
7	1941 ± 112 ^bc^	61.2 ± 1.3 ^bcde^	4.87 ± 0.54 ^ab^	nd
8	2068 ± 102 ^abc^	62.5 ± 0.8 ^ab^	5.08 ± 0.58 ^ab^	1051 ± 28 ^c^
9	2164 ± 108 ^abc^	62.5 ± 1.1 ^ab^	5.43 ± 0.57 ^ab^	907 ± 30 ^d^
10	2304 ± 110 ^ab^	53.8 ± 1.0 ^f^	3.33 ± 0.52 ^b^	1261 ± 38 ^b^
11	2082 ± 107 ^abc^	61.9 ± 1.4 ^abc^	4.96 ± 0.58 ^ab^	1458 ± 35 ^a^
12	2132 ± 100 ^abc^	61.1 ± 1.1 ^bcde^	5.42 ± 0.54 ^ab^	1258 ± 32 ^b^
13	1894 ± 101 ^bc^	66.6 ± 1.0 ^a^	6.33 ± 0.58 ^a^	1214 ± 31 ^b^
14	2007 ± 102 ^abc^	61.3 ± 1.3 ^bcd^	4.62 ± 0.54 ^ab^	1231 ± 29 ^b^

nd = not detected. ^a,b,c,d,e,f^ values in the same column with different letters differ significantly when *p* < 0.05. For formulations 1–14, review Table 1.

**Table 7:** OOT (°C) of SIPHO microencapsulated with NAE.

Microencapsulated SIPHO with NAEFormulations	OOT (°C)		
	Average	SD	U
SIPHO **	169	2	10
SIPHO + GA + MD + WPI **	181	0.4	10
8	189	<1	±11
9	187	<1	±11
10	186	1	±11
11	179	1	±10
12	185	<1	±11
13	185	1	±11
14	178	1	±10

U: expanded uncertainty for the OOT test. SD: Standard deviation. The reported expanded measurement uncertainty was calculated by multiplying the combined standard uncertainty by a coverage factor k = 2, which corresponds to an approximate level of 95% confidence under normal distribution. ** Data available on [8]. For formulations 8–14, review Table 1.

## Data Availability

All related data and methods are presented in this paper. Additional inquiries should be addressed to the corresponding author.
